# Effects of Trauma-Hemorrhage and IL-6 Deficiency on Splenic Immune Function in a Murine Trauma Model

**DOI:** 10.1155/2012/186709

**Published:** 2012-02-09

**Authors:** P. Mommsen, T. Barkhausen, C. Zeckey, H. Andruszkow, C. Krettek, C. Neunaber

**Affiliations:** Trauma Department, Hannover Medical School, Carl-Neuberg-Straße 1, 30625 Hannover, Germany

## Abstract

Splenic immune function is known to be depressed following hemorrhage. The present study investigates the effects of femoral shaft fracture, isolated or in combination with hemorrhage, on early stage cytokine production capacity of splenocytes and observes the role of IL-6 under these conditions. Male IL-6 knockout (IL-6^−/−^) and wild-type mice (WT) were randomly divided into three groups: sham (S), isolated femoral fracture (Fx), and femoral fracture + volume controlled hemorrhage (TH-Fx) (*n* = 6 per group). Animals were sacrificed four hours after induction of hemorrhage and fracture. Cytokine release (TNF-*α*, IL-6, and IL-10) of isolated and LPS-stimulated splenocytes was determined by cytometric bead array. Femoral fracture with or without hemorrhage caused a suppression of in vitro cytokine production capacity of splenocytes at an early posttraumatic stage in WT and IL-6^−/−^. In the absence of IL-6, the profile of splenic cytokine secretion is significantly altered, identifying this cytokine as a potential therapeutic target to modulate the posttraumatic immune response.

## 1. Introduction

The posttraumatic immune response is characterized by a complex set of pro- and anti-inflammatory reactions in order to restore homeostasis [1] and was shown to be organ and cell specific [2, 3]. Splenic immune response and cellular immunity are known to be depressed after trauma-hemorrhage leading to an increased susceptibility to infectious complications [4, 5]. Several studies have demonstrated that splenocyte functions, such as proliferative and cytokine production capacity as well as macrophage antigen presentation function are suppressed following trauma hemorrhage [6–9]. In regards to T-cell function, a posttraumatic shift from splenic Th1 cytokine (IL-2, IFN-*γ*, and TNF-*α*) to Th2 cytokine (IL-4, IL-5, IL-10) production has been reported [10–15]. These splenic immune alterations are well described in rodent trauma models consisting of laparotomy and hemorrhagic shock [3, 16–19] and appear already 2 hours after insult induction [12, 15, 20–23]. Simple hemorrhage and laparotomy alone were demonstrated to result in a marked depression of splenic immune function, and no differences were seen in the extent of depression in the early stage, if these two insults were combined [24]. However, a prolonged splenic immunodepression of 7 days was seen after combined trauma hemorrhage [25], whereas splenic immune suppression only persists up to 3 days following isolated hemorrhage or laparotomy [26]. With a later onset at day 7, splenic immune alterations following femoral fracture and hemorrhage seem to differ from the aforementioned trauma models [27]. However, results are inconsistent due to different study designs (e.g., animal gender, genetic strain, and protocol of fracture induction). Data regarding the role of isolated femur fracture in early stage splenic immune alterations are lacking, but are of special clinical interest as femoral fracture itself as well as the stabilization by femoral nailing seems to have a significant impact on the inflammatory response and the susceptibility to infectious complications [28]. Furthermore, it is still controversially discussed whether Interleukin (IL)-6, produced by many different cell types (e.g., macrophages, endothelial, and T cells), exerts pro- or anti-inflammatory effects in the posttraumatic immune response. IL-6 seems to have both, pro- and inflammatory effects, depending on whether it is acting in a paracrine or endocrine manner [29]. Additionally, the effects of IL-6 seem to be influenced by the stimulus and the model of inflammation used in experimental studies [30]. The aim of the present pilot study was to investigate whether a femoral fracture with or without hemorrhagic shock has an impact on the splenic cytokine production capacity at an early posttraumatic stage (4 hours) and to observe the effects of IL-6 under these conditions.

## 2. Materials and Methods

### 2.1. Animal Care

Prior to initiation, the study was approved by the animal welfare committee of the state of Lower Saxony. The experiments were performed in 18 male C57Bl/6 IL-6 knockout (IL-6^−/−^) mice aged 8–10 weeks and weighing 22.0 ± 3.0 g. As a control 18 male C57Bl/6 mice of similar weight (wild type; WT) were used. The animals were bred and raised under specific pathogen-free conditions in the central animal facility of our institution. Throughout the study period, pelleted mouse feed (Altromin 1324) and water were available ad libitum. Lighting was maintained on a 12-hour cycle and temperature at 20°C ± 2°C. WT and IL-6^−/−^ animals were randomly assigned into sham groups (S) (each of 6 animals, only anaesthesia) and 2 experimental groups, in which either an isolated femur fracture or a combination of hemorrhage and femoral fracture were induced.

### 2.2. Induction of Haemorrhage and Femur Fracture

All procedures were performed after deeply anesthetizing the animals with 2.2 ± 0.3 mg of ketamine (Ketanest 100 mg/kgBW) and 0.33 ± 0.045 mg of xylazine (Rompun 15 mg/kgBW). Anesthesia was maintained during the entire study period.

A standardized femur fracture was induced in experimental groups using a blunt guillotine device with a weight of 500 g as previously described [31]. This resulted in an A-type femoral fracture combined with a moderate soft-tissue injury. A hemorrhagic shock was induced by withdrawing 60% of the total blood volume (body weight (in g) x 0.04 mL) via puncturing the orbital plexus. Resuscitation using sterile ringer's lactate was performed with four times the shed blood volume in the tail vein after 1 hour. After resuscitation, splint fixation of the femoral fracture was performed.

Sham animals were only anaesthetized without performing any surgical procedure.

### 2.3. Harvesting Procedure and Preparation of Splenocyte Cultures

Experimental animals were sacrificed four hours after induction of hemorrhage and fracture, whereas sham mice were four hours after the first anaesthesia. The spleen was removed aseptically and processed as follows.

Splenocytes were isolated as previously described [32]. In brief, spleens were gently ground between frosted microscope slides to produce a single cell suspension. This suspension was centrifuged at 300 × g for 10 min at 4°C. The erythrocytes were lysed with lysis buffer and the remaining cells were washed with PBS by centrifugation (300 × g, 15 min, 4°C). After centrifugation, cells were resuspended in RPMI 1640 (Gibco) containing 10% heat inactivated FBS and antibiotics (50 U/mL penicillin, 50 *μ*g/mL streptomycin and 5 *μ*g/mL gentamycin, all from Gibco) to get a final concentration of 1×10^6^ cells/mL. The splenocytes were then cultured in the presence of 10 *μ*g/mL LPS (LPS from *E.coli* 0111:B4, Sigma-Aldrich Inc., Steinheim, Germany) at 37°C, 95% humidity, and 5% CO_2_ for 24 hours. After incubation, the cell free suspension was collected and stored at −80°C until further analysis.

### 2.4. Flow Cytometry

Cytokine concentrations in cell-free supernatants were determined with cytokine Bead Array inflammatory kits using flow cytometry according to the manufacturer's instructions (Bender MedSystems, Vienna, Austria).

### 2.5. Statistics

Statistical analysis was performed using SPSS computer software (SPSS 11.5, Chicago, IL). Statistical significance was assumed where probability values of less than 0.05 were obtained. Comparisons between groups were performed using one-way analysis of variances (ANOVA), Student's *t*-test or the rank-sum test (Mann-Whitney *U*-test). Results are expressed as mean ± standard error of the mean (SEM).

## 3. Results

**Figure 1:**
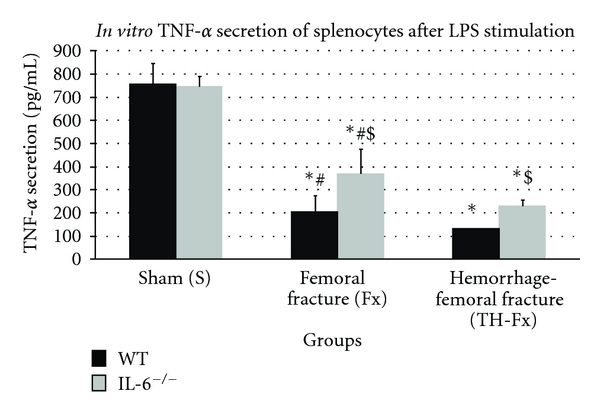
TNF-*α* productive capacity of splenocytes in response to their stimulation with LPS in wild-type (WT) and IL-6 knockout (IL-6^−/−^) mice in sham animals (group S), after an isolated femoral fracture (group Fx) and after the combination of a femoral fracture and hemorrhage (group TH-Fx). *stat. significance (*P* < 0.05) S vs. Fx and TH-Fx, ^#^stat. significance (*P* < 0.05) Fx versus. TH-Fx, ^$^stat. significance (*P* < 0.05) WT vs. IL-6^−/−^.

The *TNF-*α** secretion of stimulated splenocytes was significantly reduced following isolated femoral fracture in WT and in IL-6^−/−^ mice compared to sham animals (Figure 1). In IL-6^−/−^ mice, the decrease of the TNF-*α* release was significantly attenuated in comparison to WT animals (*P* < 0.05). In sham groups, no significant differences concerning the TNF-*α* secretion were observed between WT and IL-6^−/−^ mice (*P* > 0.05). Combined hemorrhage and femoral fracture resulted in a further significant decrease of the TNF-*α* release in WT as well as in IL-6^−/−^ mice compared to animals with an isolated femoral fracture (*P* < 0.05). Again, a significantly attenuated depression of the TNF-*α* production was demonstrated in IL-6^−/−^ mice compared to WT animals (*P* < 0.05) as shown in Figure 1.

**Figure 2:**
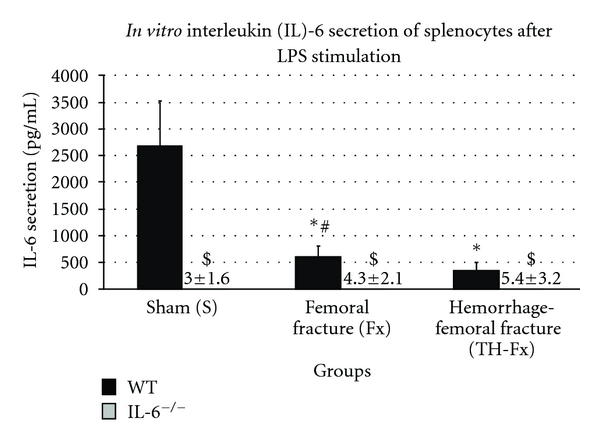
IL-6 productive capacity of splenocytes in response to their stimulation with LPS in wild-type (WT) and IL-6 knockout (IL-6^−/−^) mice in sham animals (group S), after an isolated femoral fracture (group Fx) and after the combination of a femoral fracture and hemorrhage (group TH-Fx). *stat. significance (*P* < 0.05) S vs. Fx and TH-Fx, ^#^stat. significance (*P* < 0.05) Fx versus. TH-Fx, ^$^stat. significance (*P* < 0.05) WT versus. IL-6^−/−^.

 The *IL-6* productive capacity of stimulated splenocytes was significantly decreased after femoral fracture compared to sham animals (*P* < 0.05). The combination of hemorrhagic insult and femoral fracture resulted in an additional significant suppression of the IL-6 release (*P* < 0.05) as shown in Figure 2. In IL-6^−/−^ mice, the mean IL-6 secretion was <6 pg/mL in all study groups due to the lacking IL-6 gene. Therefore, no significant differences in IL-6 release between different study groups were observed in IL-6^−/−^ mice (*P* > 0.05).

**Figure 3:**
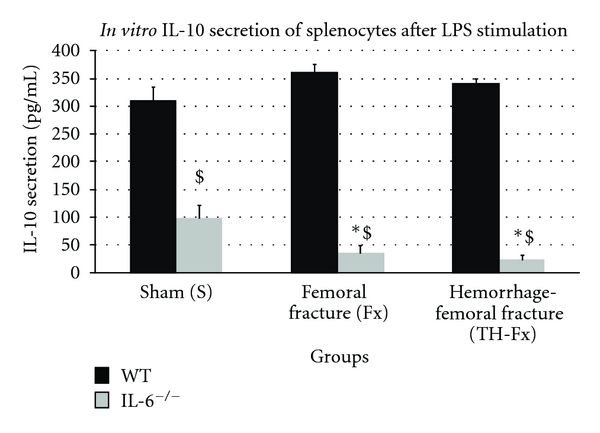
IL-10 productive capacity of splenocytes in response to their stimulation with LPS in wild-type (WT) and IL-6 knockout (IL-6^−/−^) mice in sham animals (group S), after an isolated femoral fracture (group Fx) and after the combination of a femoral fracture and hemorrhage (group TH-Fx). *stat. significance (*P* < 0.05) S versus. Fx and TH-Fx, ^$^stat. significance (*P* < 0.05) WT versus. IL-6^−/−^.

In WT mice, femoral fracture with or without hemorrhagic shock did not result in a significant increase of the IL-10 production of splenocytes compared to sham animals (*P* > 0.05). In IL-6^−/−^ mice, the IL-10 release of stimulated splenocytes was significantly decreased in all study groups compared to WT animals (Figure 3). Thereby, the IL-10 production was significantly decreased following femoral fracture with or without hemorrhagic shock compared to the sham group (*P* < 0.05). No significant differences concerning the IL-10 release were demonstrated between IL-6^−/−^ mice with an isolated femoral fracture and animals with an additional hemorrhagic shock (*P* > 0.05).

## 4. Discussion

Trauma results in a significant impairment of splenic immune functions which has been supposed to increase the susceptibility to septic complications as a result of depressed cellular immunity [33–37]. Operative stabilization of major fractures (e.g., femoral fractures) is supposed to additively enhance the trauma-induced inflammatory changes [38, 39], which has resulted in an ongoing discussion about the optimal timing of definitive fracture stabilization.

Despite the important role of the spleen in the immune response after trauma, the effects of a femoral fracture as an isolated injury or in combination with hemorrhagic shock on the early-stage splenic cytokine production has yet not been investigated. Furthermore, the role of IL-6 within these splenic immune alterations has not been elucidated. In this context, it is well described that systemic cytokines are mainly released by Kupffer cells as the largest population of tissue macrophages after trauma hemorrhage and infectious stimulus [2, 40]. As the pathophysiological role of splenic IL-6 has been discussed controversially, we focused on the importance of splenocytes and locally generated IL-6 in the posttraumatic inflammatory response.

The main findings of the present study may be summarized as follows.

1. An isolated femoral fracture resulted in a significantly depressed cytokine production capacity of splenocytes at an early stage of the posttraumatic course. The depression of splenic cytokine release was enhanced in case of an additional hemorrhagic shock. These alterations were observed in WT and IL-6^−/−^ mice.
2. The splenic immunodepression in terms of reduced TNF-*α* secretion of splenocytes was significantly attenuated in the absence of IL-6.
3. The production capacity of splenocytes for IL-10 was suppressed in the absence of IL-6 in all study groups. In contrast to WT animals, splenic IL-10 release was significantly decreased following femoral fracture with and without hemorrhagic shock.

In the present study, we were able to show that femoral fracture with and without hemorrhage results in a marked alteration of splenic cytokine production capacity at an early posttraumatic stage (4 h). In accordance, several studies reported a splenic immunodepression 2 h and 4 h following laparotomy and hemorrhagic shock [12, 15, 20–23, 41, 42]. In contrast, splenic immune depression after femoral fracture and hemorrhage seems to appear later in the posttraumatic course. Mack et al. described that the typical splenic Th1/Th2 shift was evident at day 7, but not at day 1 after trauma hemorrhage [27]. Further studies reported comparable results with marked alterations of splenic cytokine release in response to femoral fracture and hemorrhage at day 7 after insult induction [43–46]. On the other hand, Monroy et al. [47] as well as Stapleton et al. [38] found no significant alterations in splenic cytokine secretion seven days after combined femoral fracture and hemorrhage. These inconsistent results might be explained by differences in the study design. Several studies used female animals in different estrus cycle status, which probably have a marked impact as splenic immune response exhibits a gender dimorphic pattern [10]. Additionally, we induced a closed femoral shaft fracture, which was shown have an greater impact in terms of splenic immune suppression compared to open femoral fracture used in other studies [48]. As previous studies described an association between the extent of splenic immune depression and the posttraumatic susceptibility to infectious complications [12, 13, 15, 49, 50], the enhanced depression of splenic cytokine production capacity following combined femoral fracture and hemorrhagic shock might have clinical implications in the setting of multiple trauma in terms of priority and timing of definite fracture stabilization. However, the transfer of our results to the clinical setting is limited as measuring the cytokine production capacity of splenocytes only reflects a part of the T cell (namely, CD4+ T-cells) and splenic immune function. Based on our data, we could not make any statement concerning CD8+ T-cell function and proliferation capacity of splenocytes. Furthermore, it is not possible to differentiate whether depressed cytokine release is caused by a decreased production capacity of CD4+ T-cells, splenic macrophages, and/or dendritic cells as we measured cytokine secretion in the overall splenocyte culture. As the splenic immune response after trauma hemorrhage has been reported to result in a shift from Th1 to Th2 cytokines, we would have expected an increased production capacity of splenocytes for IL-10. In contrast, we only observed a nonsignificant increase of splenic IL-10 release following femoral fracture and hemorrhage in WT animals. This might be explained by the fact that we used C57BL6 mice, which were shown to be hyporesponsive to trauma hemorrhage compared to animals of the C3H/HeN strain used in other studies [19]. Moreover, it might be argued that femoral fracture and hemorrhage does not result in a Th1/Th2 shift because of the missing effect on the Th2 cytokine release in our study. In this context, it might be suggested that the depression of splenic TNF-*α* and IL-6 release results from a decreased production capacity of splenic macrophages rather than CD4+ T cells. In order to clarify this point separate isolation of splenic macrophages and/or assessment of more CD4+ T-cell-specific mediators (IL-2, INF-*γ*, IL-4, and IL-5) would have been helpful. Furthermore, significant differences in the splenic IL-10 release may not become apparent already 4 hours after trauma hemorrhage. Also for answering the question when the splenic immune function is restored after femoral fracture and hemorrhage, further studies with additional time points (e.g., 24 hours, day, 3 days, and 7 days) are needed.

Based on our results, it could be assumed that IL-6 might exert anti-inflammatory effects on the splenic immune function after trauma-hemorrhage. In the present study, we observed the principle depressive effect of femoral fracture and hemorrhagic shock on the splenic TNF-*α* release in WT and IL-6^−/−^animals. However, the depression of cytokine release was significantly attenuated in the absence of IL-6. The increased TNF-*α* levels might be explained by a potentially anti-inflammatory role of IL-6 in order to restore TNF-*α* production (negative feedback mechanism), which itself has been described as a potent inductor of IL-6 synthesis [51, 52]. The significance of this observation on immune competent cells in other tissue compartments as well as on the incidence of infectious complications and outcome after trauma has to be clarified in further studies. In the present study, the secretion of IL-10 was significantly lower in IL-6^−/−^ mice in all study groups, again pointing towards an anti-inflammatory effect of IL-6 on splenocytes, possibly by inducing IL-10 synthesis. Similar results were reported by Yang et al. for mucosal cells, where only a little or no increase of IL-10 mRNA was observed in IL-6^−/−^ mice after hemorrhage, whereas a marked increase in WT mice was found [29]. In the same study, systemic IL-10 levels were found to be comparable between WT and IL-6^−/−^ mice [29]. In contrast, after a septic insult significantly increased IL-10 plasma levels were observed in IL-6^−/−^ mice compared to WT animals [53]. Due to the results of the present studies and the observations made in the literature it might be assumed that the kind of the initiating inflammatory stimulus as well as the observed body compartment might be essential for the net effect of IL-6 [29]. It has been postulated that the net effect of IL-6 on the host inflammatory response reflects a balance of two opposing effects, a paracrine effect of IL-6 that promotes inflammation, and an endocrine effect of IL-6 that diminishes inflammation [54]. As IL-6^−/−^ mice were used in the present study, the results have to be interpreted carefully. In IL-6^−/−^ mice, there might be a functional redundancy in the regulation of the immune response following a traumatic insult [1, 55, 56]. In IL-6-deficient mice, other proinflammatory mediators may take over effects of IL-6 resulting in an only partially impaired inflammatory response compared to WT animals [55]. Furthermore, the present study is limited by the fact that FACS analyses of cell free supernatants were done only once. This may lead to a significant variation of data as shown by the standard deviations in the present study.

## 5. Conclusions

The depression of splenic cytokine production capacity after femoral fracture with and without hemorrhagic shock at an early posttraumatic stage underlines the importance of adequate treatment strategies and identification of high risk patients in order to prevent posttraumatic complications. The absence of IL-6 results in significant changes of posttraumatic splenic cytokine release indicating that the posttraumatic modulation of IL-6 synthesis might be a potential target for therapeutic interventions. However, further studies especially with an additional infectious stimulus are needed to identify the relevance of such therapies.
